# Dual-Energy Computed Tomography-Based Radiomics to Predict Peritoneal Metastasis in Gastric Cancer

**DOI:** 10.3389/fonc.2021.659981

**Published:** 2021-05-14

**Authors:** Yong Chen, Wenqi Xi, Weiwu Yao, Lingyun Wang, Zhihan Xu, Michael Wels, Fei Yuan, Chao Yan, Huan Zhang

**Affiliations:** ^1^ Department of Radiology, Ruijin Hospital, Shanghai Jiao Tong University School of Medicine, Shanghai, China; ^2^ Department of Oncology, Ruijin Hospital, Shanghai Jiao Tong University School of Medicine, Shanghai, China; ^3^ Department of Radiology, Tongren Hospital, Shanghai Jiao Tong University School of Medicine, Shanghai, China; ^4^ Department of DI CT Collaboration, Siemens Healthineers Ltd, Shanghai, China; ^5^ Department of Diagnostic Imaging Computed Tomography Image Analytics, Siemens Healthcare GmbH, Forchheim, Germany; ^6^ Department of Pathology, Ruijin Hospital, Shanghai Jiao Tong University School of Medicine, Shanghai, China; ^7^ Department of Surgery, Shanghai Key Laboratory of Gastric Neoplasms, Shanghai Institute of Digestive Surgery, Ruijin Hospital, Shanghai Jiao Tong University School of Medicine, Shanghai, China

**Keywords:** dual-energy computed tomography, iodine uptake, peritoneal metastasis, gastric cancer, radiomics

## Abstract

**Objective:**

To develop and validate a dual-energy computed tomography (DECT) derived radiomics model to predict peritoneal metastasis (PM) in patients with gastric cancer (GC).

**Methods:**

This retrospective study recruited 239 GC (non-PM = 174, PM = 65) patients with histopathological confirmation for peritoneal status from January 2015 to December 2019. All patients were randomly divided into a training cohort (n = 160) and a testing cohort (n = 79). Standardized iodine-uptake (IU) images and 120-kV-equivalent mixed images (simulating conventional CT images) from portal-venous and delayed phases were used for analysis. Two regions of interest (ROIs) including the peritoneal area and the primary tumor were independently delineated. Subsequently, 1691 and 1226 radiomics features were extracted from the peritoneal area and the primary tumor from IU and mixed images on each phase. Boruta and Spearman correlation analysis were used for feature selection. Three radiomics models were established, including the R_IU model for IU images, the R_MIX model for mixed images and the combined radiomics model (the R_comb model). Random forest was used to tune the optimal radiomics model. The performance of the clinical model and human experts to assess PM was also recorded.

**Results:**

Fourteen and three radiomics features with low redundancy and high importance were extracted from the IU and mixed images, respectively. The R_IU model showed significantly better performance to predict PM than the R_MIX model in the training cohort (AUC, 0.981 vs. 0.917, p = 0.034). No improvement was observed in the R_comb model (AUC = 0.967). The R_IU model was the optimal radiomics model which showed no overfitting in the testing cohort (AUC = 0.967, p = 0.528). The R_IU model demonstrated significantly higher predictive value on peritoneal status than the clinical model and human experts in the testing cohort (AUC, 0.785, p = 0.005; AUC, 0.732, p <0.001, respectively).

**Conclusion:**

DECT derived radiomics could serve as a non-invasive and easy-to-use biomarker to preoperatively predict PM for GC, providing opportunity for those patients to tailor appropriate treatment.

## Introduction

Gastric cancer (GC) is a serious health problem worldwide, accounting for estimated 783,000 deaths in 2018 (1). Peritoneal dissemination is the most frequent metastasis for GC. Even for patients receiving radical surgery, peritoneal metastasis (PM) occurs in up to 50% of patients, with median overall survival of 5 to 16 months (2–4). For patients with PM, upfront resection is not recommended for its uncertain benefit (5). New treatment strategies, such as intraperitoneal chemotherapy and extensive intraoperative peritoneal lavage, is of improved prognosis for those patients (6–9). Therefore, early and accurate detection for peritoneal metastasis is of an urgent need to adopt appropriate treatment to improve the prognosis for patients.

Currently, staging laparoscopy along with peritoneal lavage cytology is recommended to evaluate PM, especially for patients with radiographically suspicious signs (5, 10). However, due to invasiveness, limited sensitivity, and cost-effectiveness concerns, those tools are not universally applied (11, 12). Computed tomography (CT) is the cornerstone for clinically staging GC and is commonly used to detect PM. Nevertheless, low sensitivity hinders the role of CT, for a considerable proportion of cases are demonstrated as occult PM (13). Given biological heterogeneity into gastric tumors, a full understanding of PM is under exploration and no clinical or pathological characteristics could accurately present peritoneal spread (2). Therefore, it is of significant benefit to improve the detection of PM if a non-invasive, easy-to-use and representative tool is developed.

One vital shortcoming for multi-detector CT is its limited power to discriminate elemental compositions in materials, which share the same CT values due to its single-energy imaging. On the contrary, dual-energy computed tomography (DECT) allows quantification of different densities in mixed materials by obtaining two different energy levels (14). Applications of DECT in GC have helped to evaluate tumor invasion depth and stage migration rate, predicting lymph node metastasis and tumor response to treatment (15–18). Artificial intelligence (AI) especially deep learning has gained attention to investigate its predictive value of PM (19–21), but the interpretation is under elucidated due to its nature of “black box”. Radiomics is another emerging AI area to investigate tumor heterogeneity by extracting and analyzing high-throughput quantitative imaging features (22). Accumulated researches have explored the value of radiomics in gastric cancer, such as tumor staging, treatment response and prognosis (23–26). However, the application that integrates DECT and radiomics in GC is limited and the added value of DECT-based radiomics to detect PM is under elucidated.

PM, especially for peritoneal micro-metastasis, which is unseen by naked eyes, perhaps could be detected by DECT based on radiomics. Therefore, in this study, we aimed to develop and validate a DECT radiomics model to predict PM. For comparison, we also performed a simulated conventional CT based radiomics model. The diagnostic value of clinical factors and radiologists was also evaluated.

## Methods and Materials

### Patients

Patients were retrospectively and consecutively recruited from Ruijin Hospital (Shanghai, China) from January 2015 to December 2019. Inclusive criteria were as follows: 1) pathologically confirmed as pT3 or pT4 gastric adenocarcinoma; 2) underwent DECT scan less than one week before confirmed PM by laparoscopy, lavage cytology or surgery; 3) no previous anticancer treatment; 4) no previous abdominal surgery or peritonitis. Exclusive criteria were as follows: 1) undetected primary tumor on CT imaging; 2) unsatisfied quality of images due to insufficient stomach distention or motion artifact; 3) concurrent cancers. A total of 239 patients enrolled in this study (70 females; mean age, 62.9 years old, from 27 to 85). Patients with suspicious peritoneal implants or ascites during surgery were further evaluated by pathologists through biopsy slices or lavage cytology. The ultimate confirmation of PM relied on the consensus between involved pathologists and surgeons, according to the AJCC (American Joint Committee on Cancer) guidelines.

**Figure 1:**
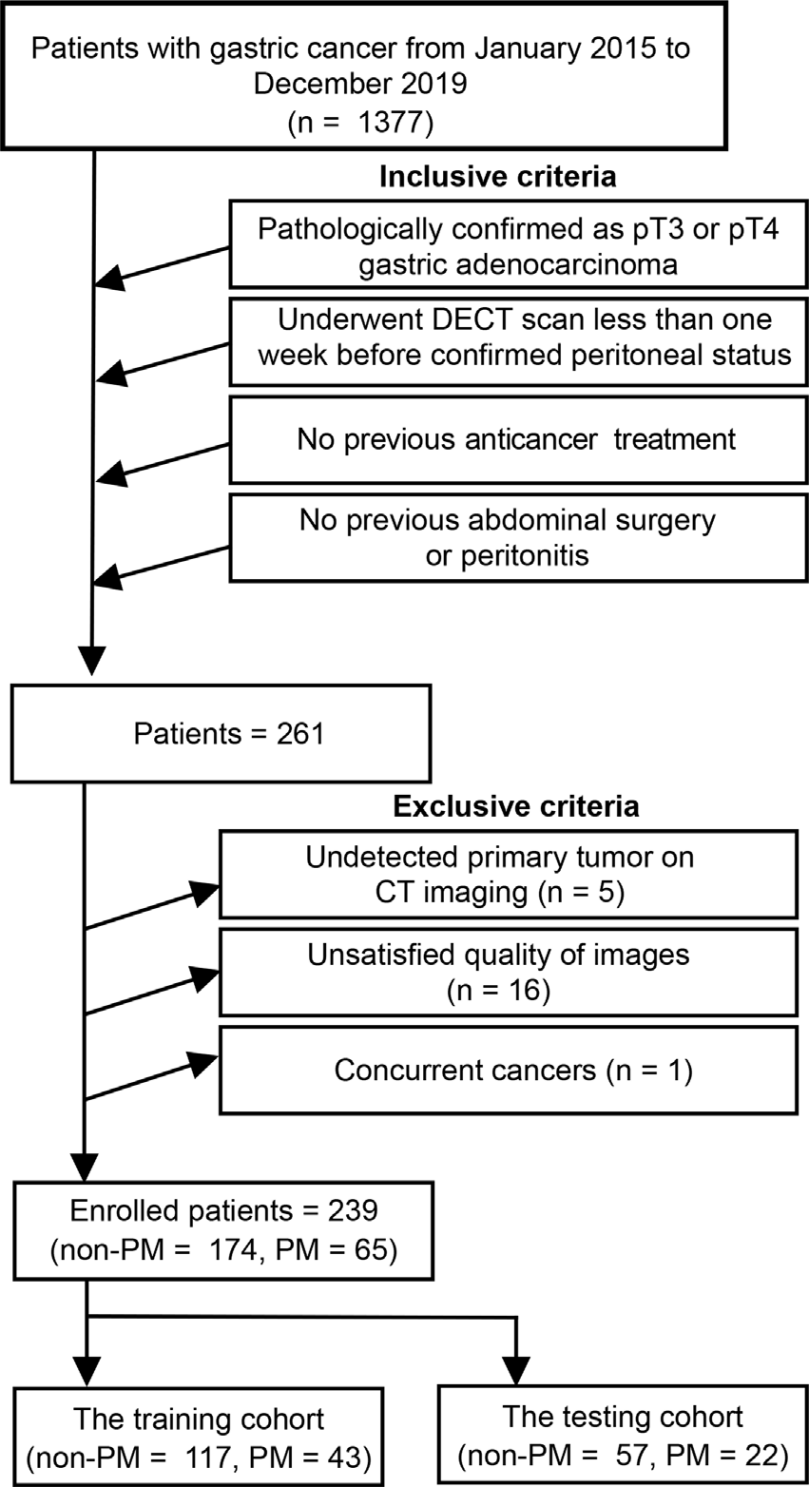
Patients enrollment in this study. DECT, dual-energy computed tomography; PM, peritoneal metastasis.

**Figure 2:**
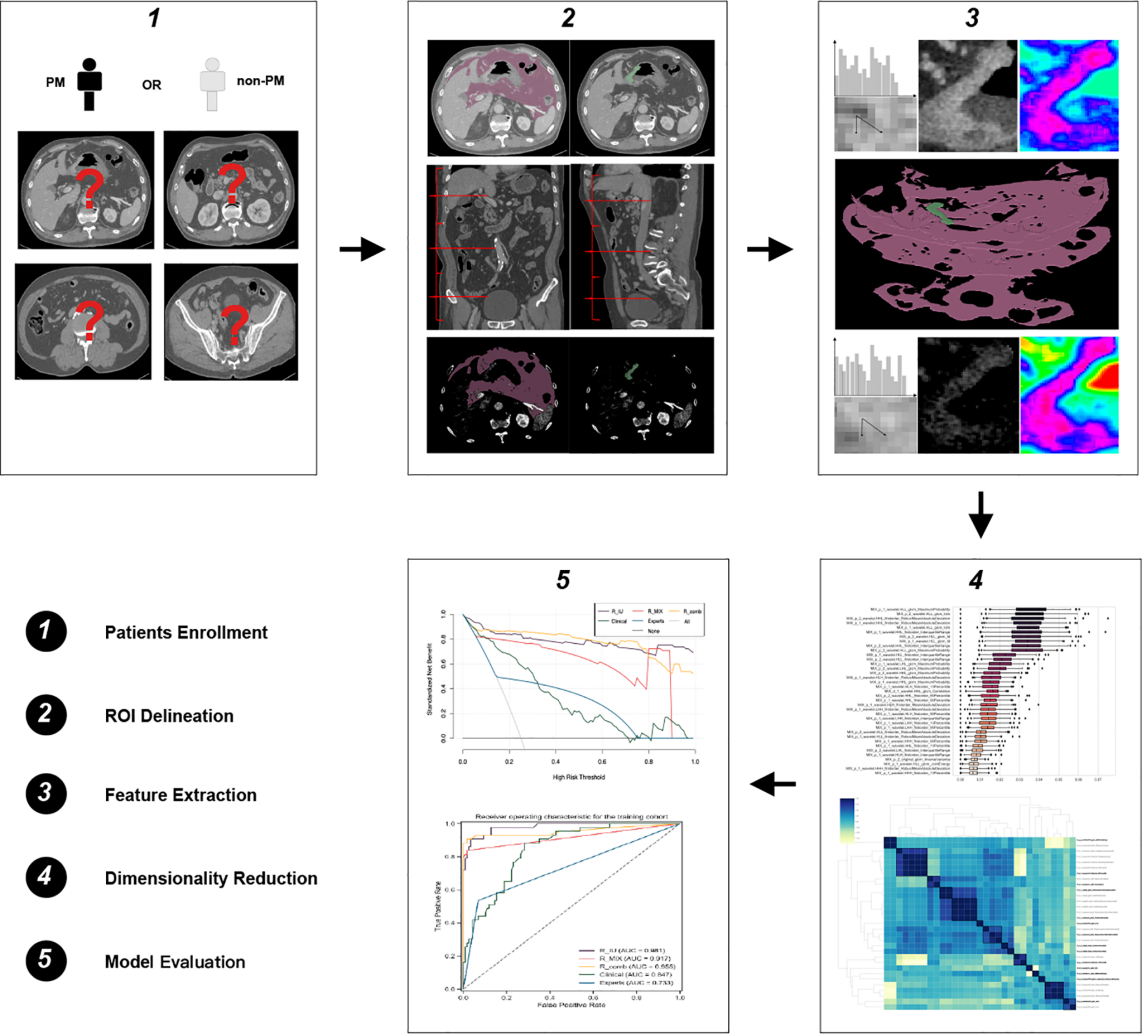
Flowchart of this study. PM, peritoneal metastasis; ROI, region of interest.

Finally, 65 patients and 174 patients were confirmed as PM and non-PM, respectively. Those patients were divided into a training cohort (n = 160) and a testing cohort (n = 79) at a ratio of 2:1 according to computer random. Baseline clinical factors included location, tumor size (cm) and depth of tumor invasion (cT) of the primary tumor, lymph node status (cN), differentiation status, Bormann type and levels of carcinoembryonic antigen (CEA), carbohydrate antigen 125 (CA125) and carbohydrate antigen 199 (CA199). The definitions for those clinical factors are implemented in **Supplementary Material**. The flowchart for patient recruitment and framework for the study are presented in **Figures 1** and **2**.

### Imaging Protocol

All patients underwent DECT scan less than one week before confirmed PM by either of two scanners (Siemens SOMATOM Definition Flash or SOMATOM Force, Siemens Healthineers) after fasting overnight. Images from portal-venous and delayed phases were processed by dual-energy software (Syngo.via, Version VB10, Siemens Healthineers) to acquisition 120-kV equivalent mixed images. The imaging protocol and dual-energy post processing were described in our previous study (27) and detailed information is presented in **Supplementary S2**. Then the images were used for post-processing by delivering to a dedicated workstation with dual-energy software (Syngo.via, Version VB20, Siemens Healthineers). Apart from the mixed images, the iodine-uptake (IU) images for portal-venous phase and delayed phase were reconstructed and obtained from the dual-energy datasets (28). The mixed images and the IU images based on both portal-venous phase and delayed phase were ultimately acquired for further delineation and analysis.

### Standardization for Iodine Concentration

For iodine concentration images, iodine quantitation was standardized in an in-house software (iodine standardization, Syngo via). The purpose of iodine standardization was to reduce the influence of technical or physiological differences resulting from variational cardiac output and phase time when loading iodine within tissues (29). A circular ROI inside the abdominal aorta was independently outlined for individuals on the portal-venous phase and delayed phase from the IU images, omitting calcified plaque secondary to atherosclerosis. The rest tissues in this image slice were used as the background to compare with the reference iodine concentration from the abdominal aorta (nIC = ICbackground/ICaorta). The standardized IU images generalized were then used for masking and analyzing.

### Segmentation

The segmentation was completed by one senior radiologist with ten years of experience in abdominal imaging, who was aware of the gastric tumor but blinded to the clinical and pathological information. The delineation was performed using an open-source software 3d-slicer (version 4.10.1) on the mixed images on the portal-venous and delayed phases, respectively. Two regions of interest (ROIs), including the peritoneal area and the primary tumor, were independently segmented. For the ROI of the primary tumor, the largest slice of the tumor on the axial image was manually segmented along with the edge of the tumor, avoiding gastric cavity and perigastric fat. For the peritoneal ROI, the delineation was performed according to abdominal regions, which consisted of the upper abdomen, the middle abdomen and the lower abdomen. The anatomical landmarks of the three regions included the upper liver, the lower costal arch, the upper sacroiliac joint and the pubic symphysis. Peritoneal ROIs were segmented at the middle slice of each abdominal region. The peritoneal area on the largest slice of the primary tumor was also included. As a candidate indicator for PM, ascites was also segmented if there was any in the peritoneal cavity. Therefore, the peritoneal ROI for each patient included four slices. The peritoneal ROI was semi-automatically segmented by setting *a priori* threshold ranging from -150HU to 50HU. The images along with the ROI were in register to the IU images to wipe off potential vessels in the peritoneal area. The ROIs delineated on the mix images were also matched to the IU images, guaranteeing they shared with the same slices. Therefore, for mixed or IU images, each had four ROIs (two on the portal-venous phase and two on the delayed phase).

### Feature Extraction

Before feature extraction, all images underwent uniform preprocessing, including resampling and filtering (see **Supplementary S3**). Radiomics features were firstly extracted from the original images and then extracted from the filtering images. Finally, there were 1691 and 1226 radiomics features extracted from the ROI of the peritoneal area and the primary tumor, respectively. All radiomics features were in accordance with the image biomarker standardization initiative (30). Detailed information on feature extraction is implemented in **Supplementary S3**.

### Reliability Analysis

We performed a reliability test to guaranteeing the reproducibility and robustness of the radiomics features. Sixty patients were randomly selected and one junior radiologist with five years of experience in abdominal imaging repeated the segmentation aforementioned. Given the same ROI of the IU and mixed images, we chose the mixed images for reliability analysis. Intra-class coefficient (ICC) was used to evaluate the reliability between the two radiologists. Radiomics features with ICCs above 0.75 were considered as good reliability.

### Dimensionality Reduction

To overcome potential overfitting, a step-wise process for feature selection was performed in the training cohort. First, Boruta was used to choose relevant features to peritoneal status. Boruta is a wrapper algorithm that selects relevant features by comparing the original importance of attributes, estimated using their permuted copies (31). To avoid randomness, the importance of each feature was calculated with 100 iterations and then a mean importance was generated. Features with the highest mean importance were defined as the confirmed features and remained. The features selected from Boruta would have highly relevant and redundant. Therefore, in the second step, we calculated the Spearman correlation coefficients for those features. Highly correlated features were clustered into the same subgroup using unsupervised clustering algorithm and feature with the highest mean importance in each subgroup was considered as the representative feature for further modeling. Consequently, features selected from the step-wise method would have high contribution and low redundancy and were included in the following modeling.

### Modeling

Radiomics features selected from IU and mixed images were independently used for modeling (the R_IU model and the R_MIX model) to discriminate PM and non-PM in the training cohort. To select the optimal radiomics model, we also built a combined model (the R_comb model) integrating IU and mixed features. Random forest (RF) algorithm with 10-fold cross-validation parameter tuned by grid search approach was implemented for model establishments. The performance of the radiomics models was then validated in the testing cohort. The weight of the features in each radiomics model were evaluated by importance ranking *via* the Gini impurity using RF.

### Model Comparison

The optimal radiomics model was in comparison with a clinical model constructed by clinical risk factors. The performance of human experts to assess PM was also recorded for all patients. Two senior radiologists who major in abdominal imaging for more than 10 years of experience independently reevaluated the peritoneal status based on CT imaging. They have informed the patients of GC but aware of the clinical and pathological information. The consensus between the radiologists was recorded and if there was any disagreement, the final decision was made by the discussion. The performance of the radiomics model, the clinical model, human experts was compared in both cohorts.

### Clinical Use

The goodness of fit for those models was evaluated to observe the agreement between the actual probability and the predicted probability made by those models. Decision curve analysis (DCA) was implemented to show the net clinical benefits of all models to predict peritoneal status.

### Statistics

Continuous variables were compared by independent-sample t-test or Mann-Whitney U test based on their distribution. Categorical variables were compared using χ^2^ or Fisher’s exact test. RF was tuned by Grid search with 10-fold cross validation. Discriminative metrics for peritoneal status included receiver operating characteristic (ROC) curve, area under the curve (AUC), accuracy, sensitivity, specificity, positive predictive value (PPV) and negative predictive value (NPV). 95% Confidence interval (CI) for each metrics was also recorded. AUCs comparison was examined by the Delong test. The goodness of fit for models was assessed and the deviation was evaluated by the Brier score. The consensus between the radiologists to evaluate peritoneal status was estimated by the kappa coefficient. The strength of kappa coefficients with 0 to 0.20, 0.21 to 0.40, 0.41 to 0.60, 0.61 to 0.80 and 0.81 to 1 was interpreted as slight, fair, moderate, substantial and almost perfect. All statistical analysis was performed with R (version 3.6.0) and Python (version 3.7). Package resources are listed in **Supplementary Table S1**. A significant difference was achieved if a two-side p-value < 0.05.

## Results

### Patients Baseline Information

No selective bias was found in terms of the positive rate of PM between the training cohort and the testing cohort (26.9% vs. 27.8%, p = 0.874). Age and gender also presented no significant bias between the two cohorts (p = 0.913 and 0.848, respectively). For patients in the training cohort, gender, ascites, tumor size, differentiation status, and Bormann type showed a significant difference to discriminate the peritoneal status (all p < 0.05). For patients in the testing cohort, age, ascites, tumor size, invasion of tumor depth, and differentiation status showed statistically different to predict the peritoneal status (all p < 0.05). The other clinical factors indicated no significant difference in both cohorts (see **Table 1**). Indices that showed a significant difference in the training cohort were considered as clinical risk factors and were used for modeling using the multivariate logistic regression.

**Table 1:** Baseline clinical characteristics for patients in the training and testing cohorts.

	The training cohort			The testing cohort		
	non-PM (n = 117)	PM (n =43)	*p*	non-PM (n = 57)	PM (n = 22)	*p*
**Gender**			0.005			0.322
Female	26 (22.2%)	20 (46.5%)		15 (26.3%)	9 (40.9%)	
Male	91 (77.8%)	23 (53.5%)		42 (73.7%)	13 (59.1%)	
**Age**	65.0 (59.0, 70.0)	65.0 (52.5, 69.0)	0.181	68.0 (60.0, 74.0)	53.5 (43.2, 57.0)	<0.001
**Ascites (ml)**			<0.001			<0.001
No ascites	94 (80.3%)	18 (41.9%)		48 (84.2%)	7 (31.8%)	
Ascites < 50	22 (18.8%)	19 (44.2%)		9 (15.8%)	12 (54.5%)	
Ascites > 50	1 (0.9%)	6 (14.0%)		0 (0.0%)	3 (13.6%)	
**Location**			0.140			0.073
Fundus and Cardia	15 (12.8%)	2 (4.7%)		7 (12.3%)	1 (4.6%)	
Body	23 (19.7%)	12 (27.9%)		13 (22.8%)	8 (36.4%)	
Antrum and pylorus	49 (41.9%)	13 (30.2%)		27 (47.4%)	5 (22.7%)	
Diffuse	30 (25.6%)	16 (37.2%)		10 (17.5%)	8 (36.4%)	
**Tumor size (cm)**	4.6 (3.7, 5.8)	5.5 (4.7, 7.0)	0.006	4.4 (3.6, 5.6)	6.2 (5.2, 8.4)	0.001
**Tumor invasion**			0.159			0.024
cT2	2 (1.7%)	0 (0.0%)		0 (0.0%)	1 (4.6%)	
cT3	6 (5.1%)	1 (2.3%)		4 (7.0%)	0 (0.0%)	
cT4a	80 (68.4%)	24 (55.8%)		41 (71.9%)	11 (50.0%)	
cT4b	29 (24.8%)	18 (41.9%)		12 (21.1%)	10 (45.5%)	
**Node lymph status**			0.349			0.356
cN0	22 (18.8%)	8 (18.6%)		9 (15.8%)	3 (13.6%)	
cN1	33 (28.2%)	8 (18.6%)		24 (42.1%)	6 (27.3%)	
cN2	47 (40.2%)	17 (39.5%)		18 (31.6%)	10 (45.5%)	
cN3a	11 (9.4%)	5 (11.6%)		5 (8.8%)	1 (4.6%)	
cN3b	4 (3.4%)	5 (11.6%)		1 (1.8%)	2 (9.1%)	
**Differentiation status**			0.007			0.007
High	1 (0.9%)	0 (0.0%)		0 (0.0%)	0 (0.0%)	
Medium	19 (16.2%)	0 (0.0%)		11 (19.3%)	0 (0.0%)	
Low	89 (76.1%)	41 (95.3%)		44 (77.2%)	18 (81.8%)	
SRCC	8 (6.8%)	2 (4.7%)		2 (3.5%)	4 (18.2%)	
**Bormann**			0.017			0.189
I	3 (2.6%)	2 (4.7%)		2 (3.5%)	1 (4.6%)	
II	46 (39.3%)	10 (23.3%)		22 (38.6%)	5 (22.7%)	
III	65 (55.6%)	25 (58.1%)		30 (52.6%)	12 (54.5%)	
IV	3 (2.6%)	6 (14.0%)		3 (5.3%)	4 (18.2%)	
**CEA**			0.651			1.000
Negative	103 (88.0%)	36 (83.7%)		50 (87.7%)	20 (90.9%)	
Positive	14 (12.0%)	7 (16.3%)		7 (12.3%)	2 (9.09%)	
**CA199**			0.036			0.554
Negative	58 (49.6%)	30 (69.8%)		37 (64.9%)	12 (54.5%)	
Positive	59 (50.4%)	13 (30.2%)		20 (35.1%)	10 (45.5%)	
**CA125**			0.562			1.000
Negative	74 (63.2%)	30 (69.8%)		33 (57.9%)	13 (59.1%)	
Positive	43 (36.8%)	13 (30.2%)		24 (42.1%)	9 (40.9%)	

CA125, carbohydrate antigen 125; CA199, carbohydrate antigen 199; CEA, carcinoembryonic antigen; PM, peritoneal metastasis.

### Reliability Analysis

For features extracted from the portal-venous phase, 213 and 138 radiomics features showed robust with an ICC higher than 0.75 for the peritoneal area and the primary tumor, respectively. As for features from the delayed phase, 523 and 189 radiomics features showed robust with an ICC higher than 0.75 for the peritoneal and the primary tumor, respectively. Therefore, for the IU and mixed images, each had 1063 reliable radiomics features for further analysis.

### Dimensionality Reduction

**Figure 3:**
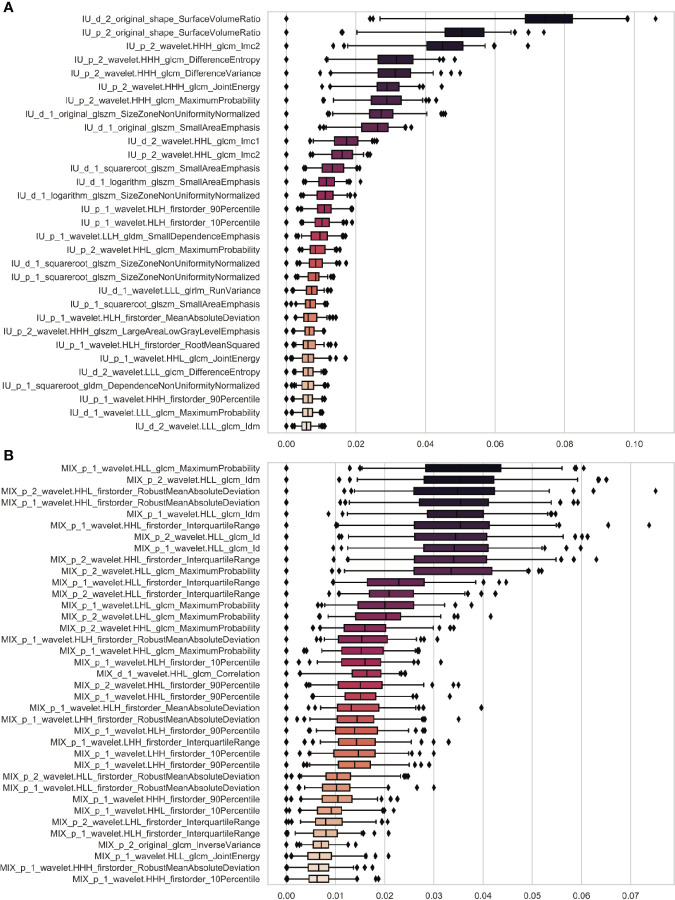
Feature selection using Boruta algorithm. The boxplots showed there were 31 and 37 confirmed radiomics features selected using Boruta algorithm from the IU images **(A)** and the mixed images **(B)**. The horizontal coordinate and vertical coordinate independently represent feature importance and feature names. The importance of features was calculated using 100 iterations. The name of each feature was defined as follows: image (IU or mixed [MIX])_phase (portal-venous [p] or delayed [d])_ROI (1, the peritoneal area; 2, the primary tumor)_preprocessing_category (firstorder, texture or shape)_feature. For example, for the first feature in **(A)**, i.e. IU_d_2_original_shape_SurfaceVolumeRatio, it means this feature (SurfaceVolumeRatio) belongs to shape category and is extracted from the primary tumor from IU images of the delayed phase without any preprocessing (original).

There were 31 and 37 confirmed features after Boruta analysis from IU and mixed images, respectively (**Figures 3A, B** **)**. Given high redundancy among those features, Spearman correlation analysis was performed. Unsupervised clustering indicated 14 and 3 subgroups generated for features from the IU and mixed images (**Figures S1** and **S2**). Finally, 14 and 3 features were selected for each image set by the mean importance of each feature. After tuned by RF, all the 14 radiomics features from IU images were used for RF modeling to construct the R_IU model. Similarly, the three features from mixed images were finally used to establish the R_MIX model through the RF algorithm.

To build the R_comb model, we further integrated the 14 features from the IU images with the three features from the mixed images. The correlation coefficients of those 17 features were also calculated and no redundancy was observed (**Figure S3** **)**. After tuned by RF, eight features were considered as important and used for modeling the R_comb model.

**Figure 4:**
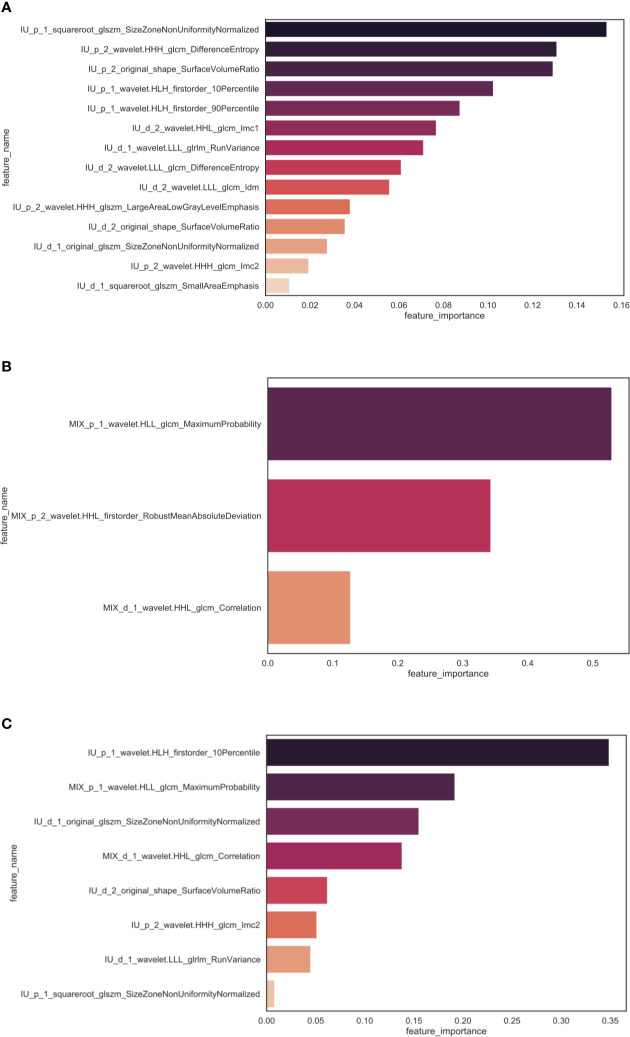
The importance ranking of features selected in each model. There were 14, 3 and 8 radiomics features selected for the R_IU model **(A)**, the R_MIX model **(B)** and the R_comb model **(C)**. In each model, the most important feature was from peritoneal area extracted from the portal-venous phase. The naming rule for feature was described aforementioned.

Consequently, the radiomics features selected by the step-wise method showed little redundant and highly important. For each model, the most important feature was coincidentally from the peritoneal area. The importance ranking of those features in each model are implemented in **Figure 4**.

### The Performance of Radiomics Models

The R_IU model showed power ability to predict PM for the gastric tumor in the training cohort, achieving an AUC of 0.981 (95%CI, 0.961-0.995) and accuracy of 95%. It also presented a considerable sensitivity and specificity (90.7% and 96.6%, respectively). This model showed good generalization by applying it to differentiate peritoneal status in the testing cohort, with an AUC of 0.967 (95%CI, 0.931-1). No overfitting was observed for the R_IU model in the testing cohort (p = 0.528). The other discriminative metrics also demonstrated a good diagnostic value of the R_IU model in the testing cohort. Detailed information on discriminative metrics is implemented in **Table 2**.

**Table 2:** Performance of each model to discriminate peritoneal status in the training cohort.

		AUC	Accuracy	Sensitivity	Specificity	PPV	NPV
The R_IU model	The training cohort	0.981 (0.961-0.995)	95% (90.4%-97.8%)	90.7% (76.9%- 97.0%)	96.6% (91.0%- 98.9%)	90.7% (76.9%- 97.0%)	96.6% (91.0%- 98.9%)
	The testing cohort	0.967 (0.931-1)	89.8% (81.0%-95.5%)	86.4% (64.0%- 96.4%)	91.2% (80.0%- 96.7%)	79.2% (57.3%- 92.1%)	94.5% (83.9%- 98.6%)
The R_MIX model	The training cohort	0.917 (0.860-0.974)	94.4% (89.6%-97.4%	83.7% (68.7%-92.7%)	98.3% (93.3%-99.7%)	94.7% (80.9%-99.1%)	94.3% (88.1%-97.5%)
	The testing cohort	0.894 (0.800-0.988)	84.8% (75.0%–91.9%)	81.8% (59.0%–94.0%)	86.0% (73.7%–93.3%)	69.2% (48.1%–84.9%)	92.5%( 80.9%–97.6%)
The R_comb model	The training cohort	0.955 (0.904–1)	96.3% (92.0%–98.6%)	90.7% (76.9%, 97.0%)	98.3% (93.3%, 99.7%)	95.1% (82.2%, 99.2%)	96.6% (91.1%, 98.9%)
	The testing cohort	0.961 (0.918–1)	92.4% (84.2%–97.2%)	86.4%(64.0%–96.4%)	94.7% (84.5%–98.6%)	86.4% (64.0%–96.4%)	94.7% (84.5%–98.6%)
The clinical model	The training cohort	0.847 (0.786–0.909)	76.3% (68.9%–82.6%)	88.4% (74.1%–95.6%)	71.8%(62.6%–79.5%)	53.5% (41.4%–65.3%)	94.4% (86.8%–97.9%)
	The testing cohort	0.785 (0.669–0.902)	74.7% (63.6%–83.8%)	63.6% (40.8%–82.0%)	78.9% (65.8%–88.2%)	53.8% (33.7%–72.9%)	84.9% (71.9%–92.8%)
Human experts	The training cohort	0.733 (0.654–0.812)	82.5% (75.7%–88.1%)	53.5% (37.8%–68.5%)	93.2% (86.6%–96.8%)	74.2% (55.1%–87.5%)	84.5% (76.8%–90.0%)
	The testing cohort	0.732 (0.623–0.842)	83.5% (73.5%–90.9%)	50%(28.8%–71.2%)	96.5% (86.8%–99.4%)	84.6% (53.7%–97.3%)	83.3%(71.7%–91.0%)

AUC, area under the curve; NPV, negative predictive value; PPV, positive predictive value.

The R_MIX model showed inferior to the R_IU model in both cohorts. For patients in the training cohort, the R_MIX model reached an AUC of 0.917 (0.860-0.974) and an accuracy of 94.4%. For patients in the testing cohort, the R_MIX model reached an AUC and accuracy of 0.894 (0.800-0.988) and 84.8%, respectively. The R_IU model was significantly better than the R_MIX model to discriminate PM in the training cohort (p = 0.034).

The R_comb model did not improve the performance compared with the R_IU model in both cohorts, with AUCs of 0.955 (95%CI, 0.904-1) and 0.961 (95%CI, 0.918-1), respectively. Other information on discriminative metrics of the R_comb model is implemented in **Table 2**.

Overall, we considered the R_IU model as the optimal radiomics model and used it as the representative for further analysis.

### Comparison With the Clinical Model and Human Experts

**Figure 5:**
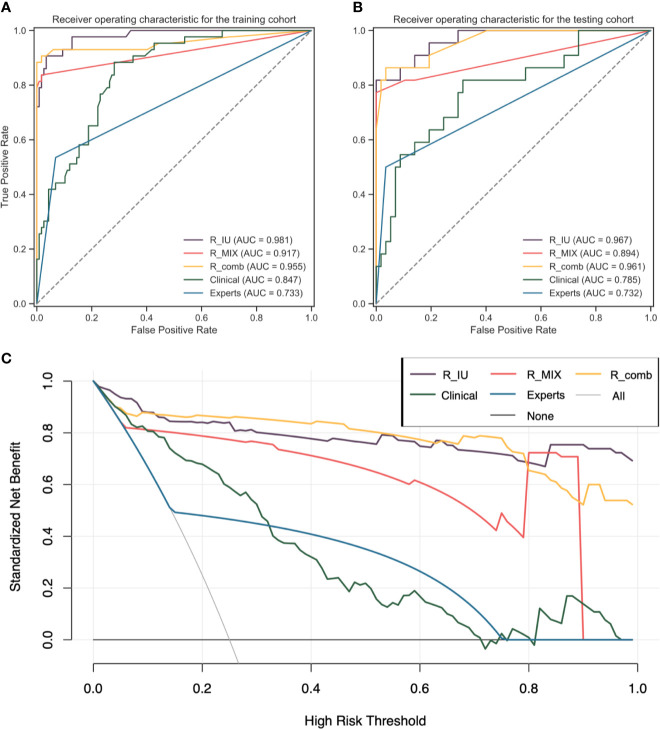
The performance of each model to predict PM for GC. **(A, B)** shows the ROC curves of each model to predict PM in the training and testing cohorts, respectively. The R_IU model performed the best in both cohorts. **(C)** shows the clinical use of each model, which demonstrated the R_IU and the R_comb models establish higher clinical benefit than other models. AUC, area under the curve; GC, gastric cancer; PM, peritoneal metastasis; ROC, receiver operating characteristic curve.

ROC curve of each model in both cohorts was visualized in **Figures 5 A, B**. Gender and ascites were indicated as independent risk factors in the multivariate logistic regression (p = 0.025 and 0.018, respectively). The clinical model showed the significantly poor performance to predict peritoneal status compared with the R_IU model in both cohorts (the training cohort, AUC: 0.847 vs. 0.981, p < 0.001; the testing cohort, AUC: 0.785 vs. 0.967, p = 0.005).

The consensus between the two human experts was almost perfect, with a kappa value of 0.83. There were 10 (5.7%) and 31 (47.7%) patients misclassified by human experts in the non-PM and PM groups, respectively. For patients in the training and testing cohorts, there were 13 and 31 patients demonstrating radiological suggestion of peritoneal metastasis, respectively. The human experts reached AUCs of 0.733 (95%CI, 0.654-0.812) and 0.732 (95%CI, 0.623-0.842) to evaluate PM in the training and testing cohorts, respectively. The sensitivity and specificity of human experts in the training cohort was 53.5% (37.8%-68.5%) and 93.2% (86.6%-96.8%), respectively. The sensitivity and specificity in the testing cohort was 50% (28.8%-71.2%) and 96.5% (86.8%-99.4%), respectively. The R_IU model showed significantly more power to differentiate peritoneal status than the human experts did in both the cohorts (both p < 0.001).

### Clinical Use and Calibration

The DCA curve showed similar good clinical benefit for patients if preoperatively diagnosing them using the R_IU model and the R_comb model, which were better than the other models (**Figure 5C**). Analysis of goodness of fit also demonstrated the R_IU model was robust to evaluate peritoneal status in both cohorts (Brier score = 0.051 and 0.055, respectively).

## Discussion

In this study, we developed and validated a DECT-derived radiomics model to predict peritoneal dissemination for GC, which outperformed the clinical model and human experts. The non-invasive and easy-to-use radiomics biomarker showed clinical benefit, which provided potentiality for GC patients with PM to avoid unnecessary surgery and adjust for new treatment strategies.

Previous studies have revealed the diagnostic value of radiomics for PM in GC based on conventional CT derived radiomics, with AUCs in the range of 0.724 to 0.873 in the validation cohort (32–34). In our study, similar performance was also observed in the R_MIX model built by 120kV-equivalent mix images in the testing cohort, with an AUC of 0.894. More importantly, our study demonstrated iodine-uptake derived radiomics provided more powerful value to predict PM than the conventional one. DECT is a candidate modality to apply in the gastric tumor due to its insight into materials decomposition (29). Our previous researches have introduced the role of DECT combined with PET/CT in dynamically monitoring the process of peritoneal metastasis for GC *in vitro* (35). In this study, we demonstrated the DECT-derived radiomics could accurately diagnosing PM for GC patients. DECT provides the possibility of materials decomposition. The R-IU model significantly outperformed the R-MIX model in the testing cohort, demonstrating the superiority of the iodine images. On the other hand, although AUC and sensitivity were not improved in the R_comb model compared with the R-IU model in the testing cohort, other discrimination metrics (i.e., accuracy, specificity, PPV and NPV) showed slightly better in the R_comb model, indicating the necessity for a synthesis of two models to predict PM in GC. The combination of materials decomposition brought from iodine images as well as mixed images and radiomics could probably detect micro-metastasis and tumor heterogeneity of GC.

The radiomics model also significantly outperformed the clinical model in diagnosing PM in both cohorts. It was reported that females were susceptible to peritoneal dissemination (36). In this study, we also found gender was an independent risk factor in the multivariable logistic regression and females were with significantly increased risk for PM. Ascites are an important predictor for PM. It was reported that the possibility of PM reached 75% to 100% if more than 50ml of ascites was manifested in CT (37, 38). In our study, ascites was an independent risk factor to indicate PM (p < 0.001) and the degree of it was strongly correlated with PM. We also found tumor size, differentiation status, Bormann type and CA199 showed significance between PM and non-PM groups in the univariable analysis but not presented as independent risk factors. The clinical model integrated those clinical and pathological parameters achieved a moderate performance to discriminate PM and non-PM, indicating limited specificity of those variables.

The peritoneal status evaluated by human experts was also unsatisfactory comparing to the R_IU model. CT is the recommended modality to preoperatively staging GC and evaluate peritoneal status with high specificity of 96.2% to 99% (13, 39). However, due to micro-metastasis undetectable in CT, the sensitivity was limited in a range of 25% to 50.9% (13, 36). In this study, we reevaluated the preoperative status of the peritoneum and similar performance was observed as previous reported (sensitivity and specificity, 50%-53.5% and 93.2%-96.5%). Nevertheless, our R_IU model was significantly better than human experts to predict PM in both cohorts, especially with improved sensitivity, probably indicating the advantage of the radiomics model to discern occult PM.

We simultaneously outlined and analyzed the primary tumor as well as the peritoneal area for the radiomics process. Notably, we segmented ROIs on 2D instead of 3D slices (which is much time-consuming), for a multi-center study has demonstrated that models built by 2D radiomic features revealed similar performance in evaluating preoperative characteristics of GC compared with those built by 3D features (40). Nevertheless, to utmost capture information of peritoneum, we delineate the peritoneal ROIs according to the anatomical distribution of the peritoneal cancer index (PCI) (41). PCI is used to assess the extent of peritoneal dissemination and is considered as a prognostic indicator for patients with peritoneal carcinomatosis, thus providing vital information for patient management and treatment tailor for patients with gastric tumor (42, 43). However, laparotomy is a must to evaluate PCI. The peritoneal ROIs were simulated as PCI, and for simplification, we chose three representative cross-sectional slices to inflect PCI. We also delineated the peritoneal area where the largest slice of the primary tumor settled, for the initial metastasis probably occurs in this area according to the “seed and soil” hypothesis (44). In the three radiomics model, the most important feature in each model was unlimitedly from the peritoneal area, indicating the unneglectable role of peritoneum in delineation and only delineating the primary tumor seems insufficient to assess PM.

Gray-level normalization for medical images is a prerequisite against discretization and for feature robustness to ensure radiomics as a reliable biomarker (45, 46). In this study, the iodine images were standardized to decrease the individual circulation variation resulting from technical reasons and patients, helping to reconstruct uniform iodine-uptake images and normalize variable gray levels. Previous studies reported the standardization of iodine concentration was beneficial to staging tumors and evaluating treatment response (47–49). Unlike standardizing iodine uptake by comparing the concentration between tumors and aorta, we compared the iodine concentration between background tissues to the aorta. This method was probably representative to normalize an individual’s iodine uptake effecting on gray-level normalization of the whole images and thus equally treated ROIs for both the primary tumor and the peritoneal regions.

Our study has several limitations. First, this is a single-center study and the sample size is limited, therefore, the generalization of the DECT derived radiomics needs further external validation. Meanwhile, the retrospective nature of the study probably introduced selective bias for patients. Second, due to time-consuming, delineation for the entire peritoneum area was abandoned, which may omit potential metastasis in the peritoneal area. Furthermore, it was worth noting that considering the real-world clinical setting, part of the patients enrolled in our study were radiologically suggestive as the peritoneal spread, which could improve the performance of the radiomics model. Besides, Lauren classification especially the diffuse subtype was considered as a risk factor for peritoneal spread (34, 50). In this study, this parameter was abandoned because it was unavailable to a few patients who were transferred to our hospital for upfront resection and the biopsy was unavailable before surgery. Our clinical model demonstrated limited power of those parameters to predict peritoneal status and the value of Lauren type to represent PM is undermined due to high tumor heterogeneity of GC (2). Lastly, the biological meaning of the radiomics features selected remain elucidation.

In summary, the DECT based radiomics model is a noninvasive, easy-to-use and representative tool to preoperatively predict PM for GC, which of significant benefit to adopt appropriate treatment to improve the prognosis for those patients.

## Data Availability Statement

The datasets generated in the study are included in the article/**Supplementary Material**. Further inquiries can be directed to the corresponding authors.

## Ethics Statement

The studies involving human participants were reviewed and approved by Ethics Committee of Ruijin Hospital. The ethics committee waived the requirement of written informed consent for participation.

## Author Contributions

Conceptualization, HZ, CY, and FY. Methodology, ZX and WX. Software, MW. Validation, YC and ZX. Formal analysis, WY. Investigation, LW. Resources, HZ and FY. Data curation, YC and WX. Writing—original draft preparation, YC. Writing—review and editing, HZ and WY. Visualization, ZX. Supervision, HZ. Project administration, FY. Funding acquisition, HZ, CY, and WY. All authors contributed to the article and approved the submitted version.

## Funding

This work was funded by Shanghai Science and Technology Commission Science and Technology Innovation Action Clinical Innovation Field (no. 18411953000), Medical Engineering Cross Research Foundation of Shanghai Jiaotong University (no. YG2019ZDB09), the National Natural Science Foundation of China (no. 81771789, 81771790 and 81772518), and Multicenter Clinical Trial of Shanghai Jiao Tong University School of Medicine (no. DLY201602). The funders had no role in the design of the study; in the collection, analyses, or interpretation of data; in the writing of the manuscript; or in the decision to publish the results.

## Conflict of Interest

ZX is employed by Siemens Healthineers Ltd. MW is employed by Siemens Healthcare GmbH.

The remaining authors declare that the research was conducted in the absence of any commercial or financial relationships that could be construed as a potential conflict of interest.

## Abbreviations

AI, artificial intelligence; AJCC, American Joint Committee on Cancer; AUC, area under the curve; CA125, carbohydrate antigen 125; CA199, carbohydrate antigen 199; CEA, carcinoembryonic antigen; CI, confidence interval; DCA, decision curve analysis; DECT, dual-energy computed tomography; GC, gastric cancer; ICC, intra-class coefficient; IU, iodine uptake; NPV, negative predictive value; PCI, peritoneal cancer index; PM, peritoneal metastasis; PPV, positive predictive value; RF, random forest; ROC, receiver operating characteristic curve; ROI, region of interest.
