# Supplementary material for: Dual-Energy Computed Tomography-Based Radiomics to Predict Peritoneal Metastasis in Gastric Cancer
Source: Front Oncol. 2021 May 14;11:659981. doi: 10.3389/fonc.2021.659981 (PMC8160383; doi:10.3389/fonc.2021.659981)
Supplement: Supplementary file 1 [file DataSheet_1.docx]

Supplementary Material

**Supplementary S1** Definition for clinical factors

Tumor size was measured by the longest diameter on cross-sectional image. Depth of tumor invasion (cT) was according to radiologically staging for the primary tumor. Lymph node status (cN) was according to radiologically evaluate the involved status for perigastric lymph nodes. Differentiation status was classified as good, moderate and poor differentiation for adenocarcinoma and signet-ring cell carcinoma (SRCC) if there were more than 50% of signet cells. The normal upper levels of serum indices for CA125, CA199 and CEA were 35u/ml, 35u/ml, and 5ng/ml. All serum indices were collected within one week before PM was confirmed by laparoscopy, lavage cytology or surgery.

**Supplementary S2** Imaging protocol

Before the examination, all patients drank 1000-1500 ml of water and were injected with 20 mg of scopolamine. The tube voltages of the DECT scans were 100 kV and Sn140 kV, with reference tube currents of 230 mA and 178 mA, respectively. Real-time tube current modulation (CARE Dose 4D, Siemens Medical Solutions) was performed when scanning with a collimator of 32 × 0.6 mm and a pitch of 0.6. After a routine nonenhanced scan, three contrast-enhanced phases (arterial, portal-venous and delayed phases) were performed following the intravenous administration of iodinated contrast material (Ultravist 370; Schering, Berlin, Germany) at 1.5 mL per kilogram at a rate of 3.5 mL/s using a pump injector. The arterial phase was determined by the time to peak enhancement of the celiac trunk, which covered the whole stomach. The portal venous phase and delayed phase then followed, with a delay time of 20 s after the arterial phase and 150 s after the administration of the contrast agents. Then, all images were anonymously retrieved.

Images from portal-venous and delayed phases were reconstructed by a D30f kernel and formed mixed 120 kV with a linear blending technique using a slice thickness of 1.5 mm.

**Supplementary S3** Imaging preprocessing and feature extraction

All images, including IU and mixed images, were resampled into a uniform voxel space of 1*1*1mm3. Then two filters, including Laplacian of Gaussian (LOG) and wavelet transformation were applied to preprocessing the images. For LOG, five sigma values, presenting the Gauss standard deviation of convolution kernel, were used (from 0.5 to 2.5, equally separating by 0.5). For wavelet transformation, high-pass or low-pass filters were simultaneously used in three dimensions and therefore eight different transformations were generated. Finally, after processing by the two filters, 13 new images were generated.

There were 110 radiomics features from three subgroups, including first-order features (n = 18), texture features (n = 75) and shape features (n = 17). Radiomics features were firstly extracted from the original images and then extracted from the filtering images. For the shape features, they are only appearance-dependency and therefore filters do not affect them. Apart from that, four types of mathematical transformations (including square, square root, exponential and logarithm) were used for first-order and texture features. Finally, for the peritoneal area in each phase of the IU or mixed images, there were 1691 (110 + 93*[5+8+4]) features extracted. However, due to the ROI of the primary tumor was a two-dimensional slice, features for LOG filters could not be calculated. Therefore, for the primary tumor, there were 1226 (110 + 93*[8+4]) radiomics features extracted.

**Supplementary Table S1** Packages used in this study

| **Statistical analysis** | **Packages** | **Version** | **Web** |
| --- | --- | --- | --- |
| Reliability test: intraclass correlation coefficient | ‘pingouin’ in Python | 0.3.9 | https://pingouin-stats.org/ |
| Feature selection: Boruta | ‘Boruta’ in Python | 0.3 | https://pypi.org/project/Boruta/ |
| Feature selection: Correlation | ‘sicpy’ in Python | 1.6.0 | https://www.scipy.org/ |
| Model establishment: Random Forest | ‘scikit-learn’ in Python | 0.13.2 | https://scikit-learn.org/stable/ |
| Model performance evaluation: Calibration curve and Brier score calculation | ‘scikit-learn’ in Python | 0.13.2 | https://scikit-learn.org/stable/ |
| Clinical model establishment: multivariate logistic regression | ‘rms’ in R | 6.0-1 | https://CRAN.R-project.org/package=rms |
| Model performance evaluation: decision curve analysis | ‘rmda’ in R | 1.6 | https://CRAN.R-project.org/package=rmda |
| Model performance evaluation: Delong Test and 95%CI for ROC curve analysis | ‘pROC’ in R | 1.16.2 | https://CRAN.R-project.org/package=pROC |

Abbreviations: CI, confidence interval; ROC, receiver operating characteristic curve.

Supplementary Figure S1 Selecting features with low redundancy for the IU images using Spearman correlation analysis


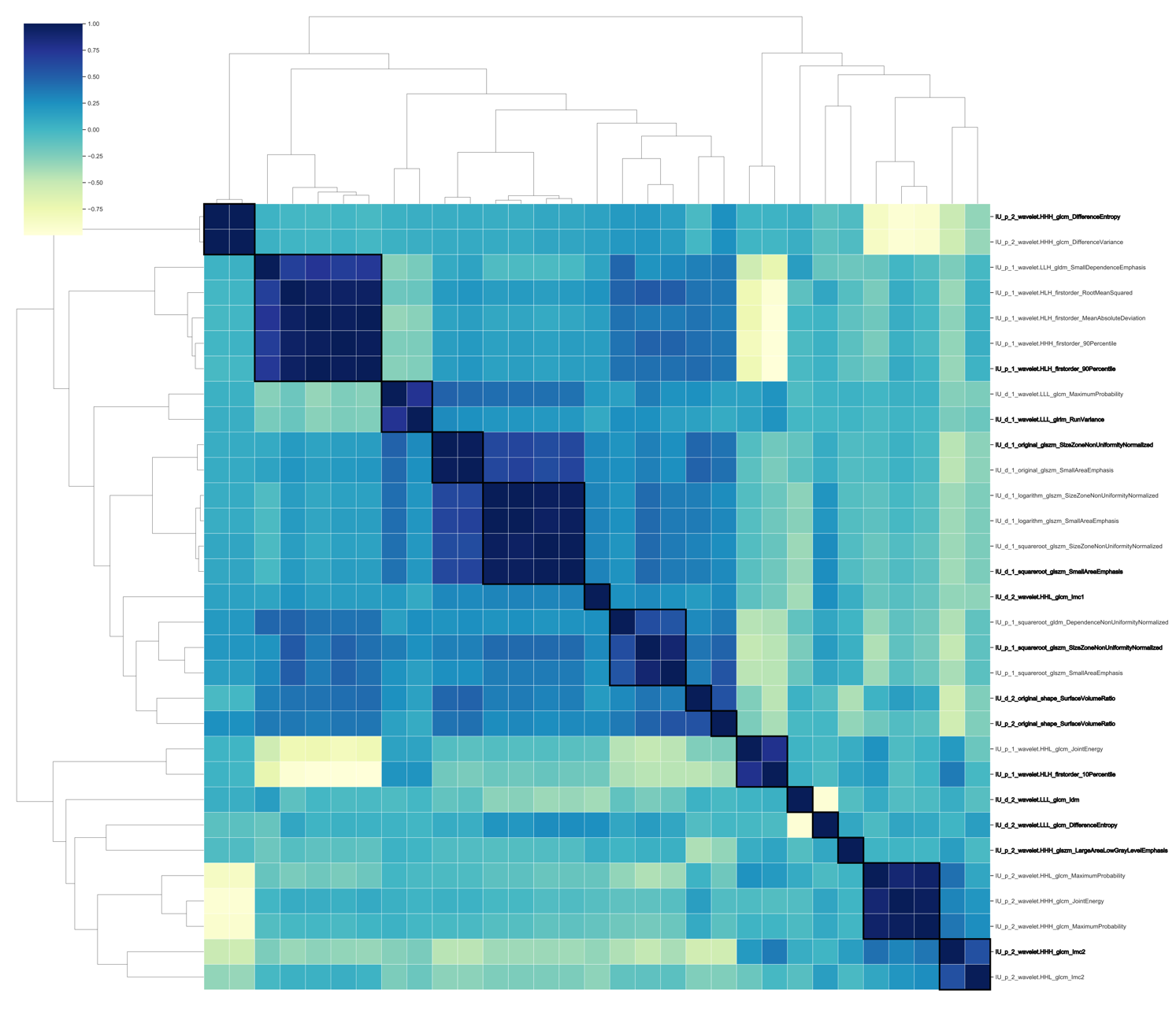


The heatmap showed the correlation of 31 radiomics features selected by Boruta algorithm for the IU images. Unsupervised clustering indicated 14 radiomics features (bold) were finally selected for the IU images by the criteria of mean importance calculated from Boruta analysis. The name of each feature was defined as follows: image (IU)_phase (portal-venous [p] or delayed [d])_ROI (1, the peritoneal area; 2, the primary tumor)_preprocessing_category (firstorder, texture or shape)_feature. For example, for the first feature ranking top, i.e., IU_p_2_waveletHHH_glcm_DifferenceEntropy, it means this feature (DifferenceEntropy) belongs to texture category (glcm subgroup) and is extracted from the primary tumor from IU images of the portal-venous phase with waveletHHH preprocessing adopted.

Supplementary Figure S2 Selecting features with low redundancy for the mixed images using Spearman correlation analysis


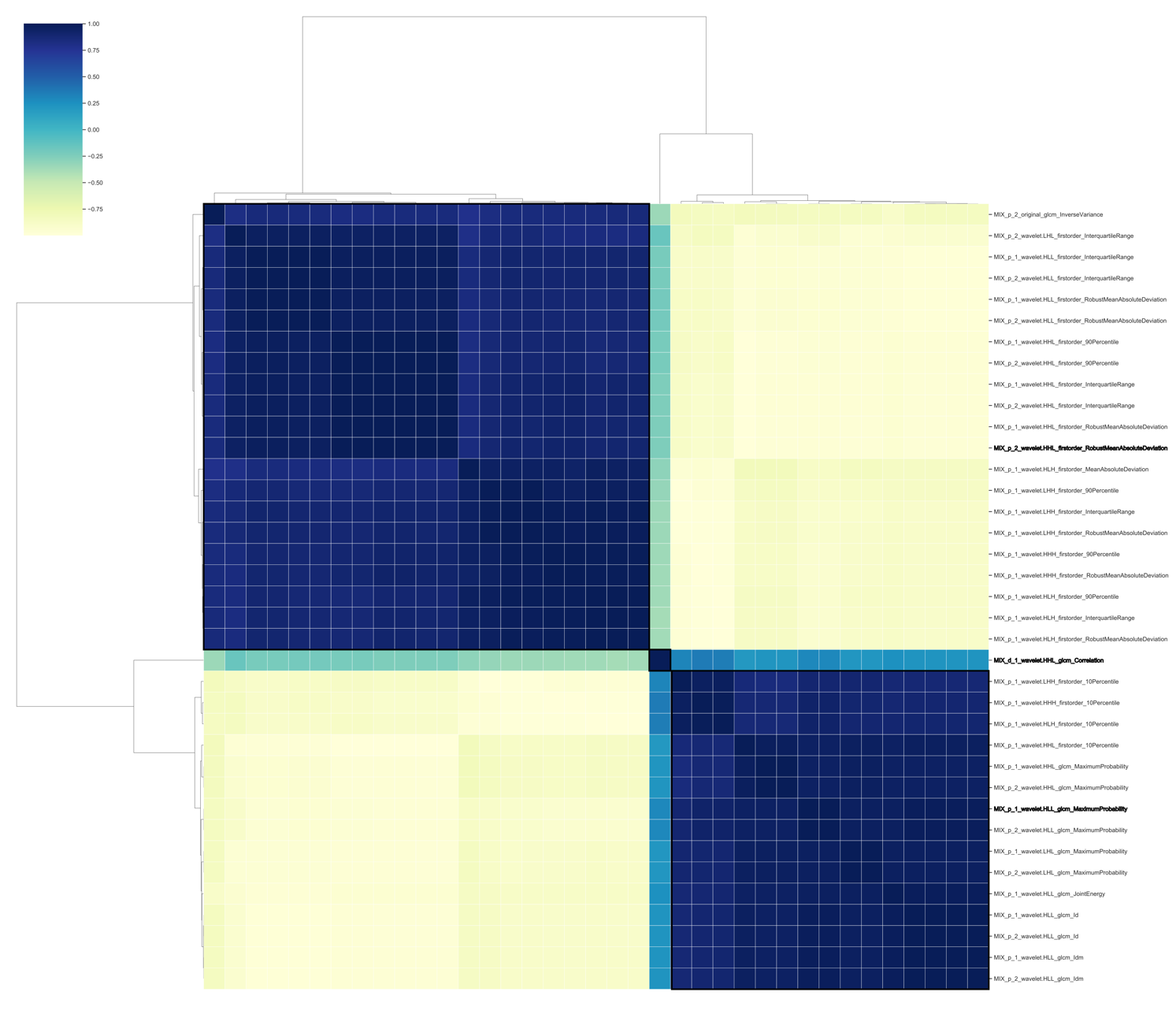


The heatmap showed the correlation of 37 radiomics features selected by Boruta algorithm for the mixed images. Unsupervised clustering indicated 3 radiomics features (bold) were finally selected for the mixed images by the criteria of mean importance calculated from Boruta analysis. The name of each feature was defined as follows: image (mixed [MIX])_phase (portal-venous [p] or delayed [d])_ROI (1, the peritoneal area; 2, the primary tumor)_preprocessing_category (firstorder, texture or shape)_feature. For example, for the first feature ranking top, i.e., MIX_p_2_original_glcm_InverseVariance, it means this feature (InverseVariance) belongs to texture category (glcm subgroup) and is extracted from the primary tumor from the mixed images of the portal-venous phase without any preprocessing (original).

Supplementary Figure S3 Selecting features with low redundancy using Spearman correlation analysis when integrating IU and mixed features


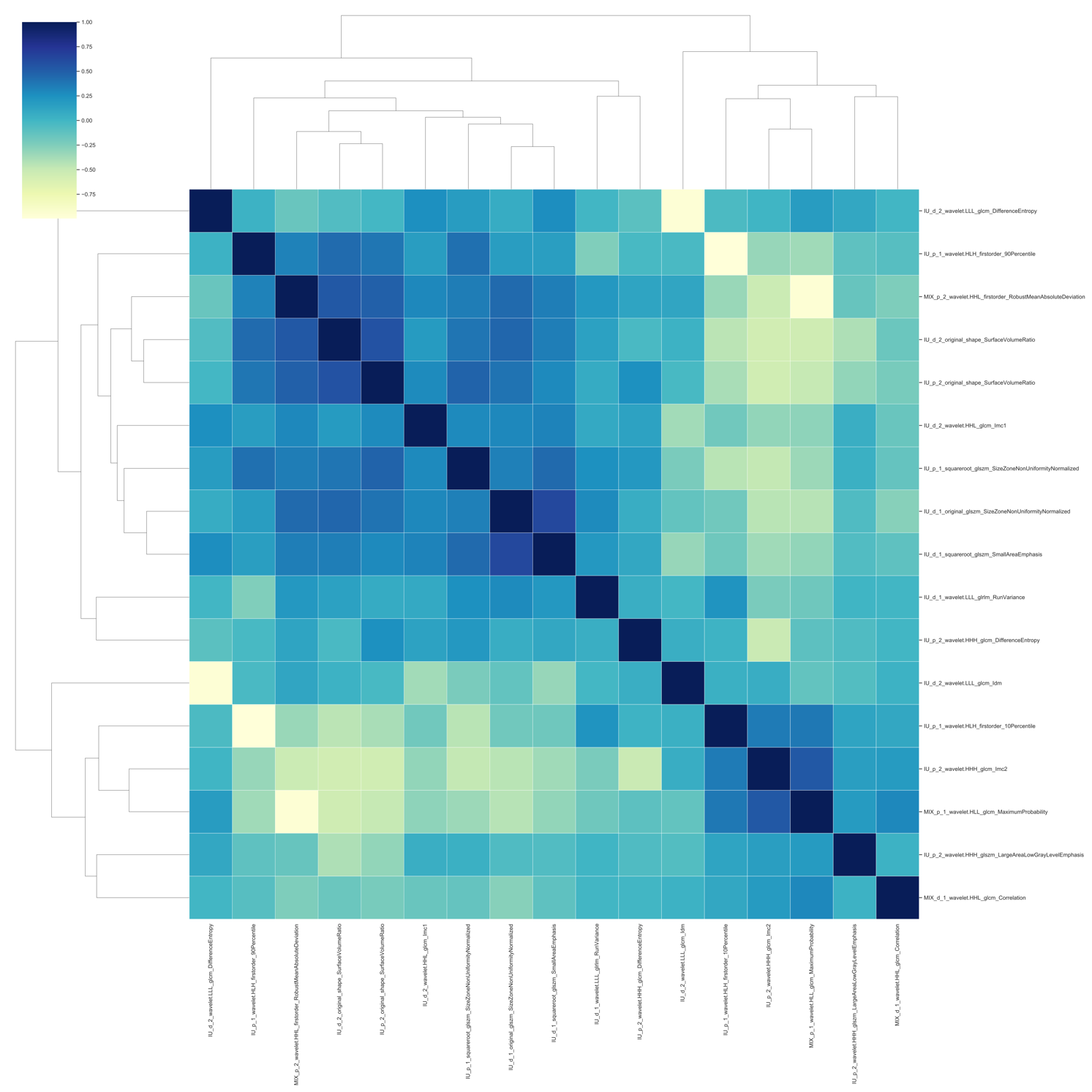


The heatmap showed the correlation of integrated 17 radiomics features from the IU and mixed images. Unsupervised clustering indicated no features were redundancy. The name of each feature was defined as follows: image (IU or mixed [MIX])_phase (portal-venous [p] or delayed [d])_ROI (1, the peritoneal area; 2, the primary tumor)_preprocessing_category (firstorder, texture or shape)_feature. For example, for the first feature ranking top, i.e., MIX_p_2_original_glcm_InverseVariance, it means this feature (InverseVariance) belongs to texture category (glcm subgroup) and is extracted from the primary tumor from the mixed images of the portal-venous phase without any preprocessing (original).
